# Ferroptosis-Related Genes Are Potential Therapeutic Targets and the Model of These Genes Influences Overall Survival of NSCLC Patients

**DOI:** 10.3390/cells11142207

**Published:** 2022-07-15

**Authors:** Na Zhang, Yangyang Wu, Yifan Wu, Lihong Wang, Jingfei Chen, Xiaosa Wang, Louisa S. Chard Dunmall, Zhenguo Cheng, Yaohe Wang

**Affiliations:** 1 National Center for International Research in Cell and Gene Therapy, Sino-British Research Centre for Molecular Oncology, State Key Laboratory of Esophageal Cancer Prevention Treatment, School of Basic Medical Sciences, Academy of Medical Sciences, Zhengzhou University, Zhenzhou 450000, China; nazhang0129@126.com (N.Z.); wuyangyang9737@163.com (Y.W.); yifanwu0805@163.com (Y.W.); lhwang2017@163.com (L.W.); ccalmokok@163.com (J.C.); wxs10829@163.com (X.W.); l.chard@qmul.ac.uk (L.S.C.D.); 2 Centre for Cancer Biomarkers & Biotherapeutics, Barts Cancer Institute, Queen Mary University of London, London EC1M 6BQ, UK

**Keywords:** ferroptosis, lung adenocarcinoma, lung squamous cell carcinoma, ferroptosis score model, drug therapy

## Abstract

Background: Lung adenocarcinoma (LUAD) and lung squamous cell carcinoma (LUSCC) are two of the most common subtypes of non-small cell lung cancer (NSCLC), with high mortality rates and rising incidence worldwide. Ferroptosis is a mode of programmed cell death caused by lipid peroxidation, the accumulation of reactive oxygen species, and is dependent on iron. The recent discovery of ferroptosis has provided new insights into tumor development, and the clinical relevance of ferroptosis for tumor therapy is being increasingly appreciated. However, its role in NSCLC remains to be explored. Methods: The clinical and molecular data for 1727 LUAD and LUSCC patients and 73 control individuals were obtained from the Gene Expression Omnibus (GEO) database and the Cancer Genome Atlas (TCGA) database. Gene expression profiles, copy number variations and somatic mutations of 57 ferroptosis-related genes in 1727 tumor samples from the four datasets were used in a univariate Cox analysis and consensus clustering analysis. The biological signatures of each pattern were identified. A ferroptosis score was generated by combining the univariate Cox regression analysis and random forest algorithm followed by principal component analysis (PCA) and further investigated for its predictive and therapeutic value in LUAD and LUSCC. Results: The expression of 57 ferroptosis-related genes in NSCLC patients differed significantly from that of normal subjects. Based on unsupervised clustering of ferroptosis-related genes, we divided all patients into three ferroptosis expression pattern groups, which showed differences in ferroptosis-associated gene expression patterns, immune cell infiltration levels, prognostic characteristics and enriched pathways. Using the differentially expressed genes in the three ferroptosis expression patterns, a set of 17 ferroptosis-related gene prognostic models was established, which clustered all patients in the cohort into a low score group and a high score group, with marked differences in prognosis (*p* < 0.001). The high ferroptosis score was significantly associated with positive response to radiotherapy (*p* < 0.001), high T stage (*p* < 0.001), high N stage (*p* < 0.001) and high-grade tumor (*p* < 0.001) characteristics. Conclusions: The 17 ferroptosis-associated genes show great potential for stratifying LUAD and LUSCC patients into high and low risk groups. Interestingly, a high ferroptosis score in LUAD patients was associated with a good prognosis, whereas a similar high ferroptosis score in LUSCC patients was associated with a poor prognosis. Familiarity with the mechanisms underlying ferroptosis and its implications for the treatment of NSCLC, as well as its effect on OS and PFS, may provide guidance and insights in developing new therapeutic targets for NSCLC.

## 1. Introduction

With incidence of cancers continuing to rise, a more comprehensive understanding of disease pathogenesis is required for developing novel targeted therapeutics to address the major health and economic burden imposed by this disease [1]. Lung cancer has the highest incidence and mortality rate of all types of cancer [2]. Non-small cell lung cancer (NSCLC) accounts for approximately 80% of lung cancer cases [3] and among NSCLC cases, LUAD and LUSCC are the predominant types in most countries, accounting for more than 75% of cases [4]. Despite the tremendous advances in lung cancer treatment, with targeted therapies and immunotherapies, the five-year survival rate remains about 55% in stage I patients [5] and drops to 1% in stage IV patients [6]. The primary treatment for early-stage lung cancer patients is surgical resection, while advanced or metastatic patients are treated with systemic therapies [7]. Recently, KEYNOTE-042 studies demonstrated that the immunotherapy pembrolizumab was superior to conventional chemotherapy in patients with advanced disease regardless of the PD-L1 tumor ratio score [8]. However, because of the highly heterogeneous nature of the disease, the majority of patients do not benefit from immunotherapy [9]. A more in-depth study of the regulatory mechanisms of immunomodulatory components and the identification of more reliable predictive biomarkers may improve immunotherapeutic efficacy in LUAD and LUSCC [10,11].

Ferroptosis is a novel type of regulated cell death with distinct characteristics as compared to apoptosis [12] and has been recently described as a novel mechanism by which cancer cell growth can be regulated [13]. Indeed, several cancers have been reported as being sensitive to therapeutic induction of ferroptosis [14]. During ferroptosis, dysfunctions in iron metabolism result in an increased intracellular level of ferrous ions. These lead to reactive oxygen species (ROS) enrichment, which promotes lipid peroxidation, leading to ferroptosis [15]. Multiple pathways exist to regulate ferroptosis in cells, including the glutathione (GSH)/glutathione peroxidase 4 (GPX4) pathway that inhibits lipid peroxidation and therefore ferroptosis [16]. These pathways have been the target of development of novel therapies to promote ferroptosis in cancer cells. For example, inactivation of GPX4 has been shown to decrease the reduction of ROS and lipid peroxides and promote cell death through ferroptosis [17,18]. The sensitivity of cancer to ferroptosis is dependent on the relative levels of GPX4 [19,20]. Ferroptosis-related gene signatures have been correlated with the prognosis of patients with head and neck squamous cell carcinoma [21] and there is growing evidence that the response of many tumors (including adrenal, pancreatic, breast, ovarian cancer, melanoma and glioma) to immunotherapy or chemotherapy may be associated with ferroptosis [22].

To date, studies on the role of ferroptosis in NSCLC are limited [23,24] and the relationship of ferroptosis to prognosis and its role in the response to therapeutic interventions remain unknown. In this study, we examined the expression profiles of ferroptosis-related genes available in public databases of NSCLC samples using functional enrichment. Ultimately, we confirmed that a ferroptosis-related gene signature correlates with patient response to drug therapy and prognosis in NSCLC patients. These data suggest that ferroptosis-related genes play an important role in the progression of NSCLC, and may provide potential prognostic markers and guidance in the development of new therapeutic targets for NSCLC.

## 2. Methods

### 2.1. Data Collection

We obtained mRNA expression profiles, copy number variants (CNVs), somatic mutations and clinical information for 585 LUAD and 550 LUSCC samples from the TCGA database through the UCSC Xena website (https://xenabrowser.net/datapages/) (accessed on 25 August 2021). We downloaded the CNV information and somatic mutations data via the “TCGA biolinks” R package. We also downloaded the GSE4573 (LUSCC) and GSE68465 (LUAD) datasets from the GEO database (https://www.ncbi.nlm.nih.gov/geo/) (accessed on 27 August 2021). All datasets included gene phenotypes, expression profiles and survival data. All expression profiles data are publicly available and follow the access policies and publication guidelines. Data preprocessing 2 (fpkm + 1) and the “sva” R package were used to normalize and further merge the expression profiles data of the four datasets in the NSCLC cohort after removing batch effects from the standardized three groups of samples. The differences in expression levels and mutations of 57 common ferroptosis-related genes between the non-small cell lung cancer samples and the control samples were analyzed. The patients were grouped according to ferroptosis-related gene expression levels using consistent clustering. Expression of the 57 ferroptosis-related genes [25] was analyzed using unsupervised cluster analysis, then divided into different ferroptosis expression patterns and further analyzed depending on disease. The stability and number of clusters were analyzed using consistent clustering algorithm by the “Consensus Cluster Plus” R package. The distance used was Euclidean distance and K-means was the clustering method used. To guarantee the stability of the classification, 1000 repetitions were performed in this analysis.

### 2.2. Gene Set Variation Analysis (GSVA) and Single-Sample Gene Set Enrichment Analysis (ssGSEA)

We performed gene set variation analysis (GSVA) via the “GSVA” R package to analyze the biological pathway differences in ferroptosis-related gene expression patterns among different ferroptosis score clusters in NSCLC. GSVA is an unsupervised, non-parametric analytical method used to determine changes in pathway and biological process activity [26]. The information regarding 60 ferroptosis-related genes is shown in Table S1 [27]. We also performed single-sample gene set enrichment analysis (ssGSEA) to evaluate the proportions, differences and infiltration ratios of 24 types of immune cells in different ferroptosis expression clusters using the “GSVA” R package. We used the Wilcoxon test to compare the ferroptosis cluster samples and performed Cox regression analysis to compare prognostic differences in different immune cells; group differences were analyzed using binary logistic regression analysis, with tumor purity corrected as the confounding factor. The Bonferroni correction was applied for multiple-comparison correction.

### 2.3. Model Establishment and Ferroptosis Score Calculation

Genes expressed differentially between different ferroptosis patterns were analyzed using the “limma” R package. The significance standard was adjusted by Benjamini and Hochberg correction and p values were set as adj.P.Val < 0.05, with fold change below 0.5 or above 2. To further analyze differential gene expression, we applied the random forest method for removing redundant genes and genes significantly associated with prognosis were extracted for further principal component analysis (PCA). The formula for calculating ferroptosis score was as follows: Ferroptosisscore = ∑ (PC1_i_ + PC2_i_), PC1_i_ and PC2_i_ are the principal components 1 and 2 of the expression level of ferroptosis-related genes. The samples were clustered into a high score group and a low score group depending on the median ferroptosis score. 

### 2.4. Correlation between Ferroptosis Score and Other Biological Processes

Gene sets associated with biological processes (such as immune checkpoints, DNA damage repair, antigen processing and presentation, epithelial mesenchymal transition, mismatch repair and nucleotide excision repairing) were constructed by Mariathasan et al. [28,29]. GSVA analysis was applied to calculate the enrichment scores of biological functions in each individual dataset. To confirm the correlation between ferroptosis scores and related biological pathways, Pearson correlation analysis of ferroptosis scores and enrichment scores for these biological processes was performed.

### 2.5. CNV Analysis

The GISTIC method based on Affymetrix SNP6 CEL files was applied for detecting the change in common copy number for all samples. The parameters for the significance level of change were set as Q ≤ 0.05, and the confidence level of peak interval determination was set as 0.95. All these analyses were performed via the corresponding MutSigCV module in an online analysis tool developed by the Broad Research Institute (https://cloud.genepattern.org/gp/pages/index.jsf) (accessed on 20 September 2021).

### 2.6. Tumor Half Maximal Inhibitory Concentration 50 (IC50)

Based on the expression profiles of the combined 2 TCGA and 2 GEO datasets, we estimated the IC50 of therapeutic drugs (erlotinib, paclitaxel, gemcitabine, imatinib and cisplatin) using the “pRRophetic” R package and compared the IC50 differences between the high ferroptosis score and low ferroptosis score subgroups.

### 2.7. Statistical Analysis

The survival differences between the high ferroptosis score group and the low ferroptosis score group were analyzed using a Wilcoxon test. We used the Kaplan–Meier method to generate the survival curves for prognostic analysis and used a log-rank test for the significance test. The prediction of a ferroptosis score by immunotherapy was evaluated through the receiver operating characteristic curve (ROC) and the “pROC” R package was used to quantify the area under the curve (AUC). The “maftools” R package was used for analysis of the mutation profiles of samples in the high and low ferroptosis score groups when the mutation profiles were displayed. The chromosomal distribution of the 57 ferroptosis genes in 23 pairs of chromosomes was analyzed using the “RCircos” R package. A *p* < 0.05 was considered to be significant.

## 3. Results

### 3.1. Patient Characteristics

**Figure 1:**
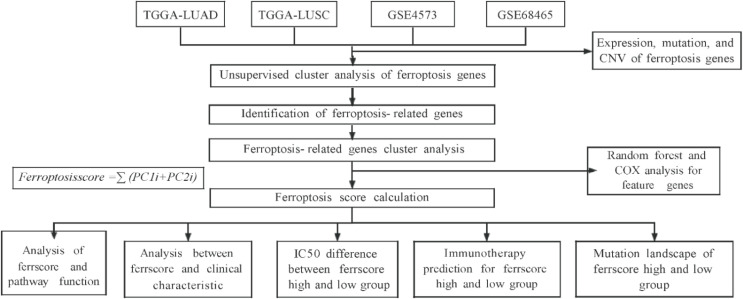
Flow chart of data collection and analysis.

A flow diagram of this study is shown in Figure 1. We extracted data for 585 LUAD patients from the TCGA-LUAD database. Data from 463 LUAD patients were obtained from the GSE68465 dataset. Data from 550 LUSCC patients were extracted from TCGA-LUSCC database and data for 130 LUSCC patients were obtained from the GSE4573 dataset. Data for controls (73) were extracted from the TCGA-LUAD and LUSCC database. The TCGA-LUAD and TCGA-LUSCC databases contain 60 ferroptosis-related genes [30] (Table S1); however, the GSE4573 and GSE68465 datasets are missing the “akr1c2”, “FANCD2” and “ZEB1” genes and, as such, only include 57 ferroptosis-related genes. There were 335 male patients and 403 female patients collected in the TCGA-LUAD database. In total, 87 patients received chemotherapy and 556 patients did not. In the TCGA-LUSCC database, 558 male patients and 207 female patients were selected. In total, 70 patients received chemotherapy and 534 did not. In the GEO dataset GSE68465, there were 223 male patients and 220 female patients. Among them, 150 were at Stage I, 251 at Stage II, 28 at Stage III and 12 at Stage IV. The LUSCC dataset GSE4573 in the GEO database contained 82 male patients and 48 female patients. Among them, 73 were at Stage I, 34 at Stage II and 23 at Stage III. The samples in each database are shown in Table 1.

### 3.2. Genetic Variations of Ferroptosis-Related Genes in LUAD and LUSCC Patients

**Figure 2:**
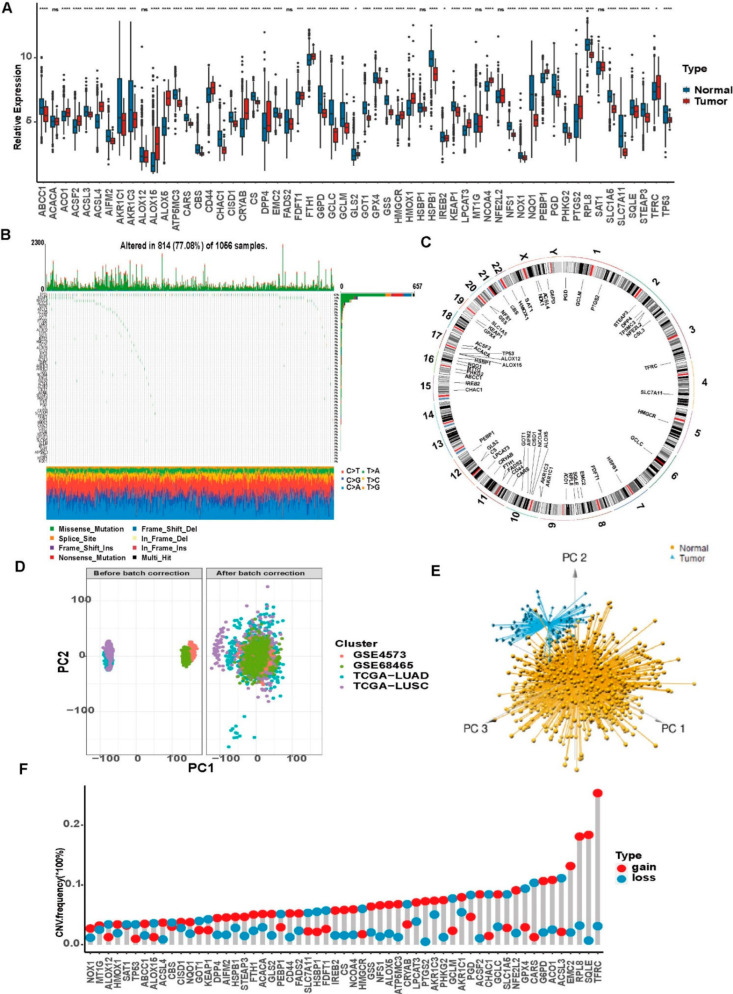
Genetic variation of ferroptosis-related genes. (**A**) The expression level of ferroptosis-related genes in the LUAD and LUSCC samples and control samples in the merged datasets. The *p* values are shown as: ns, not significant; * *p* < 0.05; *** *p* < 0.001; **** *p* < 0.0001. (**B**) Distribution and mutation types of ferroptosis-related genes in the TCGA datasets. (**C**) Localization of ferroptosis-related genes on human chromosomes. (**D**) The results of removing batch effects. (**E**) PCA of the transcriptome profiles of the three ferroptosis gene patterns. Significant differences are shown in the transcriptomes of the different ferroptosis gene patterns. (**F**) The CNV frequencies of ferroptosis-related genes in the TCGA dataset; blue indicates deletion, red indicates amplification.

CNVs, somatic mutations and expression levels of 57 ferroptosis-related genes were summarized for merged cohorts of LUAD and LUSCC in TCGA. Gene expression was significantly different in most cases between the tumor and normal tissues (Figure 2A). Among 1056 samples, 77.08% (814/1056) of patients had mutations in ferroptosis-related genes (Figure 2B). It was found that TP53 had the highest mutation frequency (62%), followed by KEAP1 at 14%, while 4 ferroptosis-related genes (ATP5MC3, CBS, HSBP1 and NQO1) did not show any mutations in these tumor samples. CNVs were identified in 57 ferroptosis genes (Figure 2B). Each autosome and sex chromosome carries different ferroptosis-related genes (Figure 2C). All data analysis is based on the data after the debatching effects. Before debatching, the four datasets were divided into four piles with large differences; after debatching, they were clustered together to eliminate the differences (Figure 2D). PCA demonstrated that samples could be grouped using the expression of these 57 ferroptosis-related genes in the TCGA dataset (Figure 2E). CN amplification was mainly associated with TFRC, SQLE, RPL8, EMC2, ACO1 and G6PD, while CN deletions were mainly associated with ACSL3 and CARS, all with frequencies greater than 10% (Figure 2F).

### 3.3. Unsupervised Clustering of Ferroptosis-Related Genes in LUAD and LUSCC Patients 

**Figure 3:**
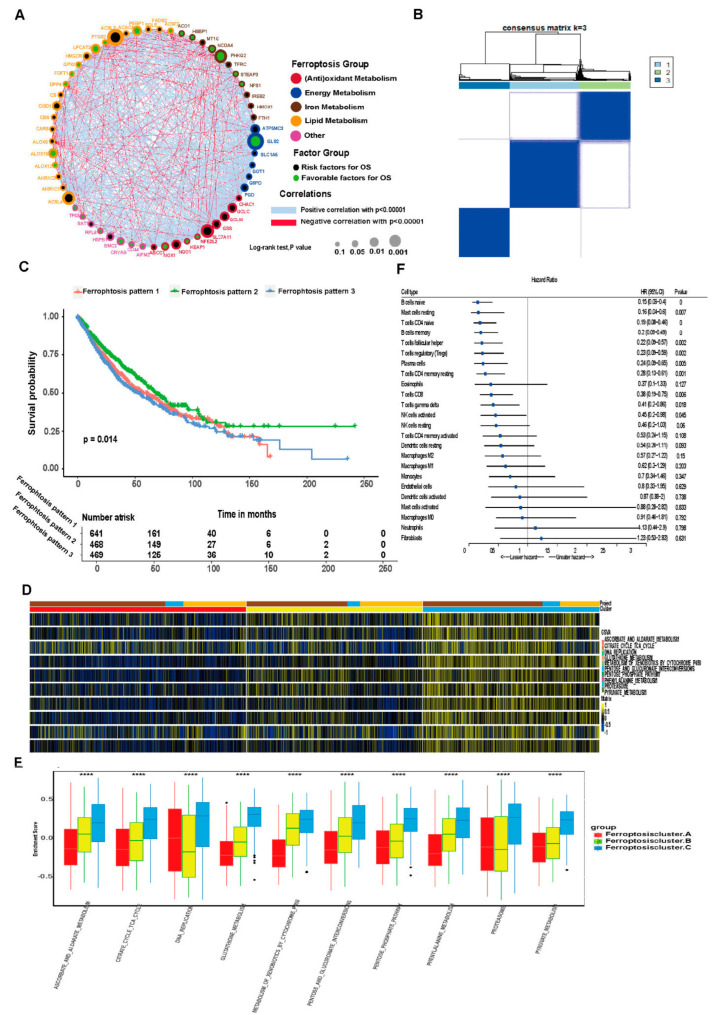
Unsupervised cluster analysis of ferroptosis-related genes in LUAD and LUSCC samples. (**A**) Interaction of ferroptosis-related genes. The size of the circles suggests the effect of each gene on predicted survival. The larger the circle area, the stronger the relationship with prognosis. Green dots inside the circles suggest protective factors, while black dots inside the circles suggest risk factors. The connecting lines between genes show their interactions. Blue indicates negative correlation and red indicates positive correlation. (**B**) Ferroptosis-related gene clustering analysis. Number 1, 2 and 3 indicate the three clusters named ferroptosis pattern 1, ferroptosis pattern 2 and ferroptosis pattern 3, respectively. (**C**) Kaplan-Meier curve shows a significant difference in survival among the three ferroptosis patterns. (**D**) GSVA shows the biological pathways activation status in different ferroptosis patterns. A heatmap was used to visualize these biological processes. Yellow indicates activation and blue indicates inhibition. (**E**) The enrichment scores of different ferroptosis patterns were significantly different in 10 pathways. (**** *p* < 0.0001). (**F**) Prognostic analysis of differential immune cell infiltration.

**Figure 4:**
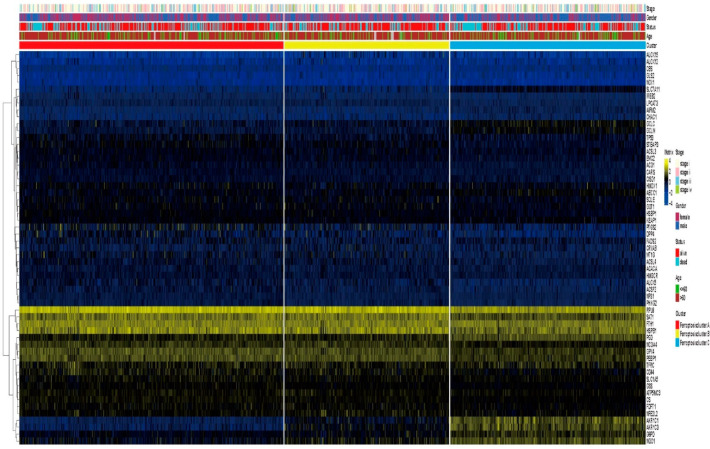
Unsupervised clustering of ferroptosis-related genes in the three ferroptosis patterns. The ferroptosis pattern, gender, tumor stage, age and survival status are used as patient annotations. Blue indicates low expression regulatory factors, yellow indicates high expression.

Univariate Cox analysis and consensus clustering analysis were applied for analyzing the expression profiles of ferroptosis-related genes from 1727 tumor samples in four datasets. Correlations between the interactions between ferroptosis genes and prognosis or regulatory mechanisms [30] such as iron metabolism, (anti)oxidative metabolism, lipid metabolism and energy metabolism are shown in Figure 3A. The consensus clustering result showed that the LUAD and LUSCC patients could be divided into three molecular subgroups: ferroptosis pattern 1, 2 and 3 (Figure 3B). There were 641 cases in pattern 1, 468 cases in pattern 2 and 469 cases in pattern 3. The three ferroptosis patterns were associated with different survival outcomes as shown in Figure 3C (*p* = 0.014). We also analyzed overall survival significances in each of two pairs. Ferroptosis patterns 1 and 3 do not show significant differences (Figure S1A–C). Ferroptosis patterns 1 and 2 do show significant differences as do ferroptosis patterns 2 and 3, demonstrating that ferroptosis-related genes could be used as a classification clustering factor. GSVA enrichment analysis was used to investigate the biological behaviors among these patterns. The results suggest that ferroptosis pattern 1 is enriched in metabolism of xenobiotics by cytochrome P450 pathways, ferroptosis cluster 2 is enriched in DNA replication pathways and ferroptosis pattern 3 is enriched in glutathione metabolism pathways (Figure 3D). The GSVA results showed that the enrichment scores of different ferroptosis patterns were significantly different in 10 pathways (Figure 3E), including ascorbate and aldarate metabolism, citrate cycle, (TCA) cycle, DNA replication, glutathione metabolism, pentose and glucuronate interconversions, metabolism of xenobiotics by cytochrome P450, proteasome, the pentose phosphate pathway, phenylalanine metabolism and pyruvate metabolism. The consensus clustering revealed significant differences in the molecular features between the three patterns. Univariate Cox analysis results of 24 immune cells with different cell ratios among the three ferroptosis patterns are shown in Figure 3F. Naïve B cells, memory B cells, naïve CD4T cells and plasma cells are protective factors in LUAD and LUSCC samples, while neutrophils and fibroblasts are risk factors in LUAD and LUSCC samples. In addition, the transcriptional profiles among the three ferroptosis patterns were significantly distinct (Figure 4). Ferroptosis pattern 1 was characterized by the increased expression of RPL8 and SAT1. RPL8 is a ferroptosis-specific mitochondrial gene and similarly to SAT1, patients with high RPL8 expression have a poorer prognosis. Ferroptosis pattern 2 is associated with high expression of GPX4 and PEBP1. Recent studies have found that PEBP1 plays a vital role in tumor development. PEBP1 induces apoptosis through TNF-α, Fas ligand, TNF-related apoptosis-inducing ligand (TRAIL) and other death ligands to promote ferroptosis. GPX4 is a key regulatory gene of ferroptosis, and decreased expression or activity induces ferroptosis. Ferroptosis pattern 3 is associated with high expression of AKR1C1, AKR1C3 and NQO1. AKR1C1 promotes lung cancer progression in an enzyme-independent manner. AKR1C3 is highly expressed in a variety of tumor cells, promoting the occurrence and development of tumors by affecting angiogenesis and similar processes and is closely related to a poor prognosis. NQO1 is the main enzyme target protein of Nrf2, regulating oxidative stress and ferroptosis in tumor cells through the Nrf2/HO-1 signaling pathway [31,32].

### 3.4. Differential Gene Expression in Ferroptosis Patterns 

**Figure 5:**
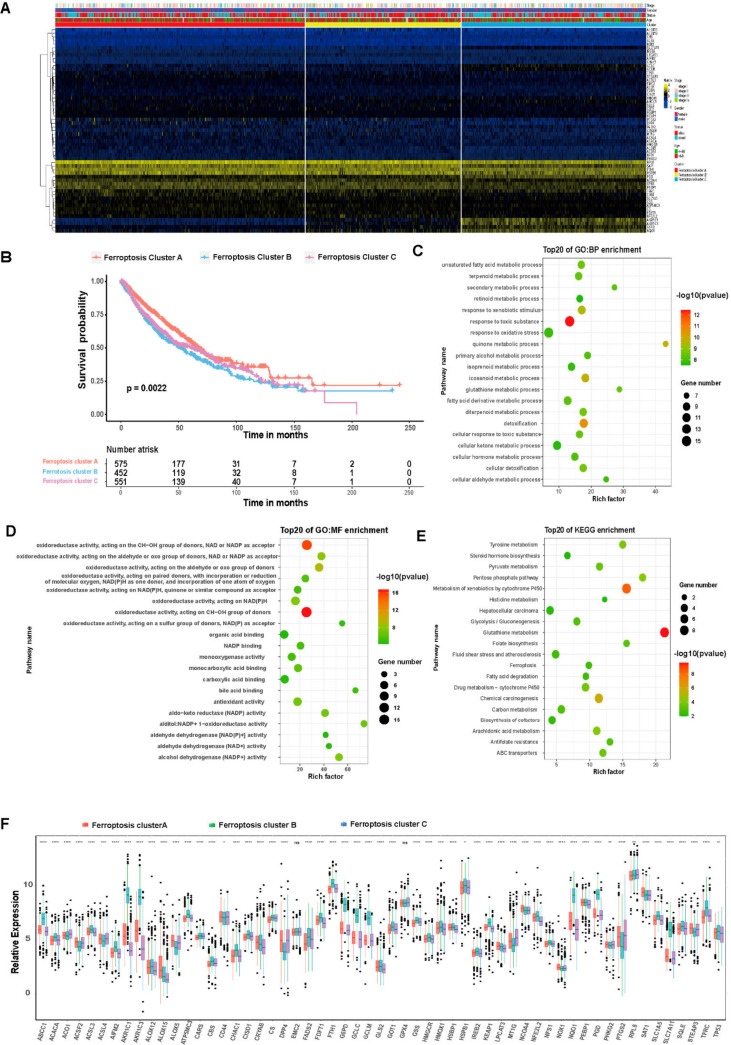
Comparison between ferroptosis-related gene cluster groups. (**A**) Unsupervised clustering analysis of ferroptosis-cluster-related genes in LUAD and LUSCC samples. The samples were grouped into three genomic subtypes named ferroptosis cluster A, B and C. (**B**) Kaplan-Meier curve for the OS in the high and low ferroptosis cluster groups. (**C**–**E**) The GO-BP, GO-MF and KEGG enrichment analyses for DEGs between the two ferroptosis gene cluster groups. (**F**) The expression of 57 ferroptosis genes in three ferroptosis cluster groups. Each row in the figure represents a GO term or a KEGG pathway. The value of the column represents enrichment score, the size of the circle stands for the number of enriched genes and the color depth represents significance from high (red) to low (green). ns, not significant; * *p* < 0.05; ** *p* < 0.01; *** *p* < 0.001, **** *p* < 0.0001.

Differential expression analysis was used to address the differences in biological effects of ferroptosis among the three ferroptosis patterns. There are 13 differentially expressed genes (DEGs) between ferroptosis pattern 1 and 2, 74 DEGs between ferroptosis pattern 1 and 3 and 47 DEGs between ferroptosis pattern 2 and 3. In total, 86 DEGs were identified (|logFC| > 1 and *p* < 0.05). These 86 DEGs (Table S3) were used to re-cluster tumor samples, and three gene clusters were obtained: ferroptosis clusters A, B and C (Figure 5A). There is a significant difference in OS prognosis among the three gene clusters (*p* = 0.0022) (Figure 5B). The 575 lung cancer patients clustered in ferroptosis cluster A were confirmed to be associated with a better prognosis. Patients associated with ferroptosis cluster C (551 patients) had a poor prognosis. The 452 patients in ferroptosis cluster B had an intermediate prognosis compared with the other two clusters. The transcriptome profiles heat map depicts the 86 most abundant DEGs identified in the three ferroptosis clusters (Figure 5A). In the three ferroptosis clusters, differences in the expression of ferroptosis-related genes were observed (N = 55), which was in accordance with the expected results of the ferroptosis patterns [33]. According to gene enrichment analysis, the DEGs were mainly involved in the response to toxic substances (Figure 5C), oxidoreductase activity (Figure 5D) and other biological processes, including metabolism of xenobiotics by cytochrome P450 and glutathione metabolism (Figure 5E). 

### 3.5. Model Establishment and Ferroptosis Score Analysis

**Figure 6:**
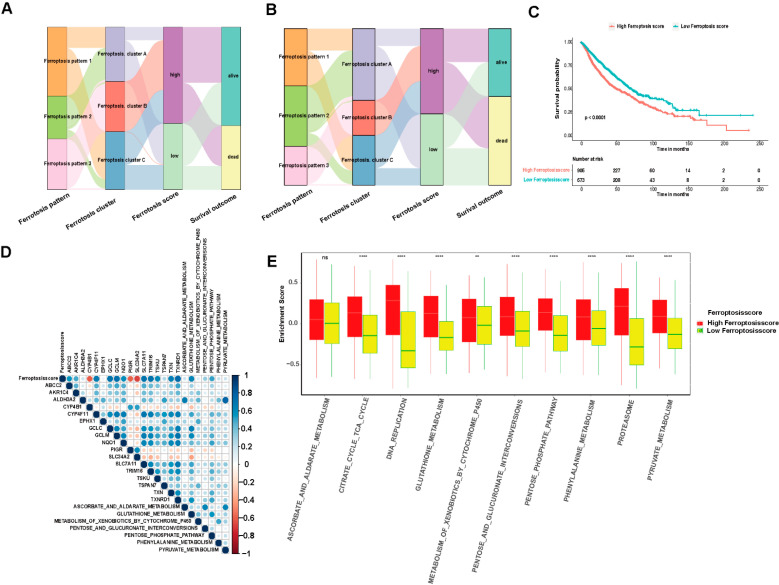
The ferroptosis score construction. (**A**,**B**). Alluvial diagram shows the ferroptosis pattern changes, including ferroptosis-related gene clusters and ferroptosis score groups and survival outcomes. (**C**) Kaplan–Meier plot for OS in the high and low ferroptosis score groups. Kaplan–Meier curves indicate that the ferroptosis score was markedly related to OS of 1578 patients in the LUAD and LUSCC cohort, of which 905 cases were in the high ferroptosis score group and 673 cases were in the low ferroptosis score group (*p* < 0.0001, log-rank test). (**D**) The correlation between the high and low ferroptosis score groups and known gene characteristics in LUAD and LUSCC. Blue represents negative correlation and red represents positive correlation. (**E**) Differences in 10 enrichment pathways between high ferroptosis score and low ferroptosis score groups (ns, not significant; ** *p* < 0.01; **** *p* < 0.0001).

**Figure 7:**
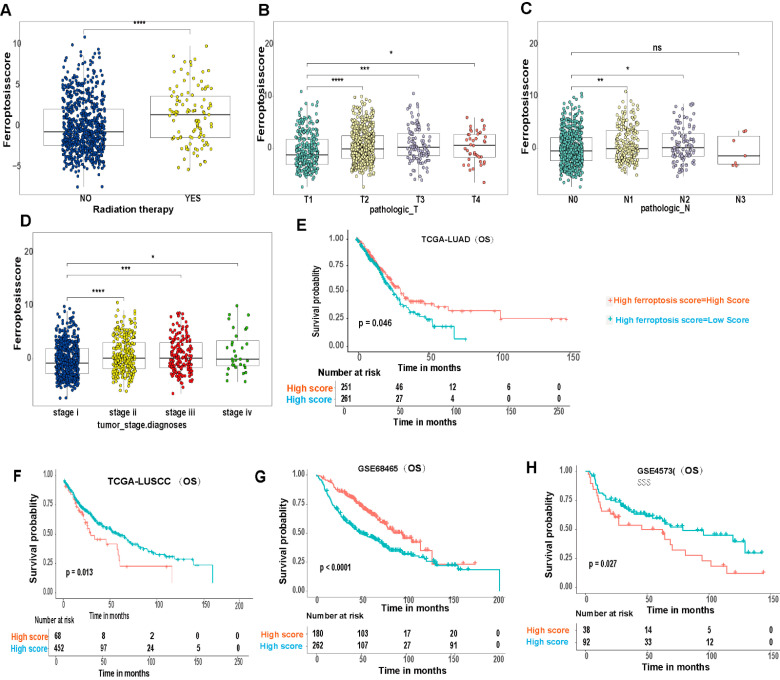
Clinical and genetic variation analyses of ferroptosis score and model validation. (**A**) The distribution of ferroptosis scores in distinct patients who did or did not receive radiation therapy in the NSCLC datasets. (**B**,**C**) The distribution of ferroptosis scores in the TN stages in the NSCLC datasets. (**D**) The distribution of ferroptosis scores across different diagnosis stages in the NSCLC datasets. (**E**) Kaplan–Meier plots for OS in the high and low ferroptosis score groups based on TCGA-LUAD. (**F**) Kaplan–Meier plots for OS in the high and low ferroptosis score group based on TCGA-LUSCC. (**G**) Kaplan–Meier plots for OS in the high ferroptosis score group and low ferroptosis score group based on GSE 68465. (**H**) Kaplan–Meier plots for OS in the high and low ferroptosis score group based on GSE4573 (* *p* < 0.05; ** *p* < 0.01; *** *p* < 0.001, **** *p* < 0.0001).

**Figure 8:**
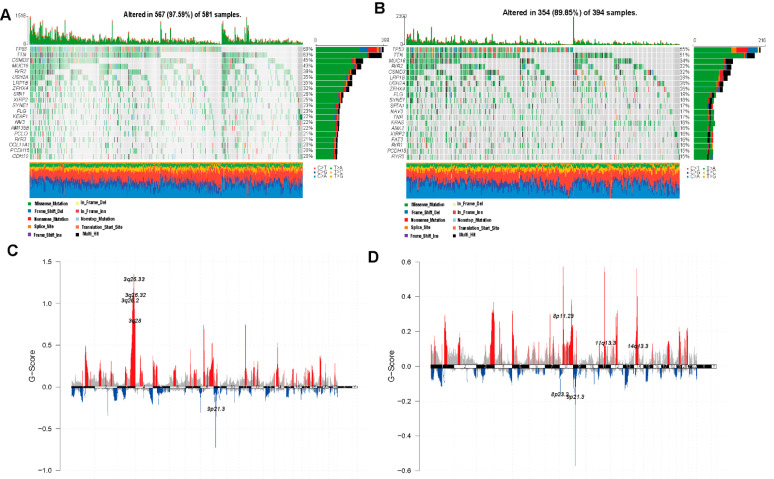
Analysis of molecular characteristics of high and low ferroptosis groups in the TCGA dataset. (**A**,**B**) The distribution of gene mutations in the samples of high (**A**) and low (**B**) ferroptosis score groups. (**C**,**D**) Distribution of copy number amplification and deletion regions in the samples of high (**C**) and low (**D**) ferroptosis score groups.

We performed prognostic analysis of 86 DEGs identified from the different ferroptosis molecular patterns (patterns 1, 2 and 3) using a univariate Cox regression model. We extracted 31 genes that have significant effects on the prognosis of patients for further analysis. We used a random forest algorithm to remove redundant genes and then conducted principal component analysis (PCA) analysis to construct a ferroptosis-relevant 17 gene signature: ABCC2, AKR1C4, ALDH3A2, CYP4B1, CYP4F11, EPHX1, GCLC, GCLM, NQO1, PIGR, SLC34A2, SLC7A11, TRIM16, TSKU, TSPAN7, TXN and TXNRD1. Both principal components 1 and 2 were selected to calculate the ferroptosis score. According to the median value of the ferroptosis score (−1.12405), the samples were clustered into the high and low ferroptosis score groups (Figure 6A,B). As shown in Figure 6C, the high ferroptosis score group was associated with a poorer prognosis and the low ferroptosis score group showed a better prognosis, which indicated that the ferroptosis score can effectively characterize prognosis for LUAD and LUSCC patients. The model showed an AUC value of 0.587 (Figure S1G). We carried out a multivariate analysis of the correlation of gene-signature risk score with outcomes among LUAD or LUSCC patients in the TCGA cohorts as shown in Table S2, (*p* < 0.05), which indicated that the ferroptosis score can serve as an independent prognostic factor. A Z value bigger than 0 for LUAD indicates that the ferroptosis score is a protective factor, and a Z value smaller than 0 for LUSCC indicates that the ferroptosis score is a risk factor. The correlation of ferroptosis scores with pathway enrichment functions (using the Pearson correlation coefficient) showed that ferroptosis scores were correlated with pathway enrichment function scores such as glutathione metabolism (Figure 6D–E), which is the major pathway regulating ferroptosis, antagonized by ferroptosis suppressor protein 1 (FSP1) and glutathione peroxidase 4 (GPX4). Figure 7A–D demonstrates that the ferroptosis score changes during radiotherapy, T stage, N stage and tumor stage. A high ferroptosis score is significantly associated with patients having received radiation therapy (*p* < 0.001), high T stage (*p* < 0.001), high N stage (*p* < 0.001) and high-grade tumor (*p* < 0.001). Interestingly, we can see that the impact of the ferroptosis score on overall survival (OS) differed in LUAD and LUSCC patients (Figure 7E–H). Using the TGCA-LUAD dataset, OS was shorter in the high score group as compared to the low score group. Conversely, using the TGCA-LUSCC dataset, OS was longer in the high score group and shorter in the low score group. Some crossover occurred in survival curves, because sudden death occurred, but there was an overall significance difference between whole datasets. Tumor copy number variation and somatic mutation landscapes were summarized for merged cohorts of LUAD and LUSCC in the TCGA database and categorized through the different ferroptosis scores. Among 581 samples with a high score, 567 (97.59%) demonstrated gene mutations, while 354 (89.95%) of 394 samples with a low score demonstrated gene mutations. TP53 and troponin (TTN) exhibited the highest mutation frequency [34] in both score groups, and the mutation frequencies were higher in the high score group (69% vs. 55% for TP53; 63% vs. 51% for TTN) (Figure 8A,B). CNV also differed between groups. The top five CNVs were located on 3q25.33, 3q26.2 in the high ferroptosis score group, 3q26.32, 3q.28 and 9p21.3, while the top five CNVs in the low ferroptosis score group were located on 8p11.23, 11q13.3, 14q13.3 and 8p23.3, 9p21.3 (Figure 8C,D).

### 3.6. Ferroptosis Score Is Linked to Therapeutic Responses in LUAD and LUSCC Patients

**Figure 9:**
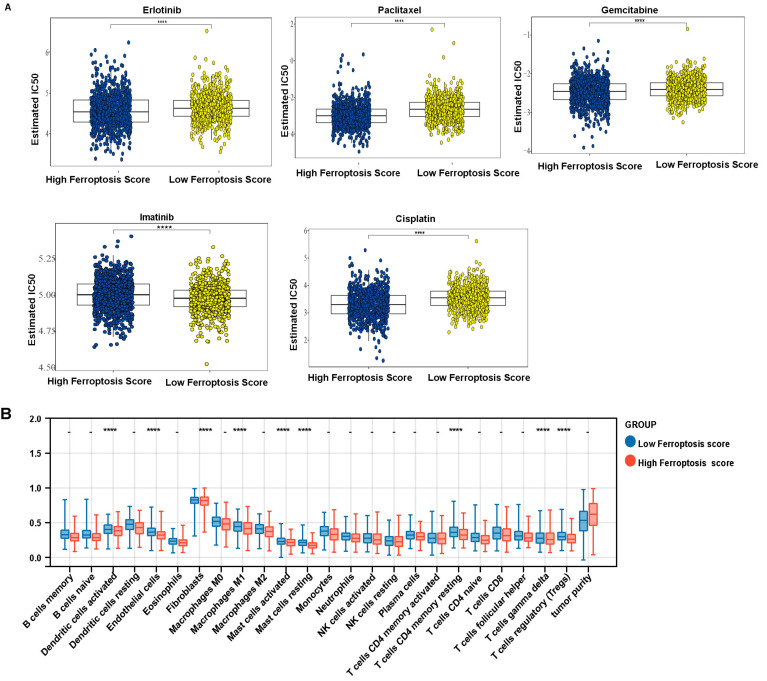
The estimated IC50 values of drugs and immune cell ratios. (**A**) The estimated IC50 values of erlotinib, paclitaxel, gemcitabine and cisplatin in the high and low ferroptosis score groups. (**B**) Immune infiltration distribution for 24 immune cells and tumor purity in two risk groups (group differences were analyzed using binary logistic regression analysis, with tumor purity corrected as the confounding factor. The Bonferroni correction was applied for multiple-comparison correction, **** *p* < 0.0001; − *p* > 0.05).

The differences in estimated IC50 value of commonly used chemotherapeutic drugs in the LUAD and LUSCC patient cohorts were compared, including first-line therapy drugs erlotinib, paclitaxel and gemcitabine and second-line therapy drugs imatinib and cisplatin (Figure 9A). The data demonstrated that the estimated IC50 value of imatinib was significantly higher in the high ferroptosis score group. Conversely, the estimated IC50 value of erlotinib, paclitaxel, gemcitabine and cisplatin was significantly lower in the high ferroptosis score groups. Group differences were analyzed using binary logistic regression analysis, with tumor purity corrected as the confounding factor. The Bonferroni correction was applied for multiple-comparison correction. Activated dendritic cells, endothelial cells, fibroblasts, macrophages M1, activated mast cells, resting mast cells, T cells CD4 memory resting, T cells gamma delta and T cells regulatory (Tregs) remained as significant different immune cells between groups (Figure 9B). These results indicate that the ferroptosis score may be immune-related and may help predict the ferroptosis subtype of NSCLC. We used the TIDE algorithm to predict the response of ferroptosis high and low score subgroups to immunotherapy (Figure S1H) and found no significant difference between the ferroptosis high and low score subgroups.

## 4. Discussion

Ferroptosis is a subtype of programmed cell death that is related to tumor regulation and response to tumor treatment [11,35]. There is a lack of systematic studies on the pathogenesis of ferroptosis, its regulatory genes and its application for prognosis and treatment in NSCLC. In this study, we explored the differences in clinical and molecular characteristics among different patient subgroups based on 57 ferroptosis-related genes. Genes were clustered into three patterns based on their expression levels to determine the association of genes with prognosis. We then used the NSCLC samples to further understand their differential expression within the three patterns and used this to calculate a ferroptosis score, creating high and low ferroptosis score groups in NSCLC samples that correlated with outcome and treatment response in patients.

The mechanism of ferroptosis involved in NSCLC tumor development and response to therapy remains unclear. In the present research, the somatic mutation rates, CNV and expression levels of 57 ferroptosis-related genes in NSCLC were initially analyzed. We found that RNA expression of 50 out of 57 ferroptosis genes was significantly different between normal and NSCLC cancer tissues. The DEGs may be used as diagnostic and prognostic markers of NSCLC. The CYP4B1 gene is a cytochrome P450 superfamily member, which is localized in the endoplasmic reticulum, mainly expressed in the human lung [36] and is necessary for ferroptosis to occur. In addition, GCLC gene expression is decreased in NSCLC tissues and this decrease can promote ferroptosis by accelerating glutathione consumption. SLC7A11 is a member of the anionic amino acid transport system and transports the anionic form of cysteine in exchange for glutamate. When cellular cysteine transport proteins are inhibited (e.g., using erastin), intracellular glutathione is depleted, causing the inactivation of GPX4. This results in increased lipid peroxidation that induces cellular ferroptosis [37]. In addition, the gene with the highest frequency of mutations associated with ferroptosis-related genes was found to be TP53, suggesting its vital function in cancer. The ascorbate and aldarate metabolism pathways interact with the citric cycle, which is the ATP production pathway required to maintain steroid hormone biosynthesis. Previous research indicated that ferroptosis occurs mainly due to altered pathways of cellular metabolism, characterized by iron-catalyzed lipid peroxidation [38,39]. GPX4 is a key gene in the regulation of apoptosis in cells [40]. In summary, ferroptosis occurs mainly due to metabolic disorders that alter intracellular reactive oxygen species, iron and polyunsaturated fatty acids. Erastin is an activator and antitumor agent of cellular permeable ferroptosis, which has three compound genes (SLC7A11, VDAC1 and VDAC2). Among them, SLC7A11 is included in our model, indicating that SLC7A11 is an important ferroptosis gene and a potential therapeutic target.

We used ssGSEA to explore the immune status between ferroptosis high and low score groups. There is significant difference in the degree of immune cell infiltration between high and low risk groups when considering both activated T and resting mast cells, activated dendritic cells, eosinophil cells, fibroblasts, microphage M1 cells, T cell CD4 memory cells, gamma delta T cells and regulatory T cells, but no significant differences when comparing the other remaining 16 cells, suggesting further targets for therapeutic intervention. In previous reports, ferroptosis in cancer cells may be a double-edged sword. On the one hand, the ferroptosis of tumor cells can release specific signaling molecules that inhibit tumor immune cells or upregulate immune checkpoints, allowing tumor cells to escape immune surveillance and keep growing. Conversely, it may release some immunogenic substances to activate the immune system and thus inhibit tumor growth [41]. Mast cells can enhance T cell-mediated immune responses, and the number of mast cells in the TME has been reported to correlate with reduced cancer progression and improved survival [42]. Dendritic cells are heterogeneous, intrinsic immune cells that can infiltrate and process tumors and play a key role in triggering antitumor T-cell immunity by providing tumor-derived antigens to naive T cells, and are major therapeutic targets for tumor immunotherapy [43]. Clinical trials have demonstrated reduced DC function in lung cancer patients [44]. Thus, immunotherapy would be beneficial for patients. We used the tumor immune dysfunction and exclusion (TIDE) algorithm to evaluate each patient’s potential response to immunotherapy, which integrates the expression signatures of T cell dysfunction and T cell exclusion to model tumor immune evasion. In our study, the TIDE showed that there is no significance difference in immunotherapy in the ferroptosis high and low score groups (Figure S1H). This result is also consistent with our analysis of immune cell infiltration. As shown in Figure 9B, activated CD4 memory T cells, naïve CD4 T cells, CD8 T cells and follicular helper T cells all do not have significance difference between ferroptosis high and low score groups. In addition, the complexity of the tumor immune microenvironment makes it harder for T-cell-related immunotherapy to exert its effectiveness. This suggests that further targets for immunotherapy intervention are needed.

By clustering gene expression levels, we found significantly different expression levels of ferroptosis-related genes between different grades and stages of NSCLC, which suggests that ferroptosis-related genes have value in the grading and diagnosis of these malignancies. We clustered genes associated with their phenotypes and found that patients with tumors showing ferroptosis cluster A had a better prognosis. This finding provides guidance for studying the prognostic relationship of specific ferroptosis-related genes with NSCLC in future. We calculated the sample ferroptosis score using differential gene expression and analyzed the prognostic relationship between NSCLC patients based on the scores. A 17-ferroptosis-associated gene prognostic model was established, which demonstrated significant benefit in diagnosis and prognosis in both LUAD and LUSCC. The high ferroptosis score group correlated with a good prognosis for LUAD, whereas, in contrast, the high ferroptosis score group correlated with a poor prognosis for LUSCC. This model has potential clinical practicability for predicting the prognosis of NSCLC patients. Of note, this study is mainly based on RNA expression data analysis; further rigorous immunohistochemistry staining for detection of the protein expression of ferroptosis-associated genes and immune cells infiltration will be more meaningful. Studying the mechanisms of ferroptosis and its biological function impact on the OS of NSCLC patients may provide guidance and insights into the stratification of high-risk patients and identification of therapeutic targets for NSCLC. Targeted therapy for NSCLC has become a successful classic case of precision medicine individualized treatment, and targeted drug therapy guided by driver mutation detection has brought considerable survival benefit to NSCLC patients. In summary, this study provides new biomarkers to predict the prognosis of NSCLC patients. However, currently, patient biopsies are not routinely genetically analyzed prior to treatment. Many hospitals are establishing genomic centers to obtain genetic information about tumors prior to treatment, and with the development of new bioinformatics technologies and the emergence of targeted drugs, new biomarkers and new assays, there is an urgent need for new testing guidelines to guide clinical practice.

## Figures and Tables

**Table 1:** Clinical characteristics of the patients involved in the study.

Characteristics		Training Cohort and Validation Cohort			
		TCGA-LUAD (878 Cases)	TCGA-LUSCC (765 Cases)	GSE4573 (130 Cases)	GSE68465 (462 Cases)
**RNA-seq cases**		585 (66.62%)	550 (71.89%)	130 (100%)	462 (100%)
**Ferroptosis gene number**		60	60	57 (missing “AKR1C2”,”FANC D2”,”ZEB1”)	57 (missing “AKR1C2”,”FANC D2”,”ZEB1”)
**SNV Cases number**		567 (64.59%)	492 (64.31%)	0	0
**CNV Cases number**		555 (63.21)	524 (68.50%)	0	0
**Cases with phenotypes**		877 (99.89%)	765 (100%)	130 (100%)	462 (100%)
**Cases with survival information**		738 (84.05%)	757 (98.95%)	130 (100%)	462 (100%)
**Gender**	Female	409 (46.58%)	207 (20.06%)	48 (36.92%)	220 (47.62%)
	Male	469 (53.43%)	558 (72.94%)	82 (63.08%)	242 (52.38%)
**Age**	<65	330 (37.59)	258 (33.73%)	31 (23.85%)	214 (46.32%)
	≥65	402 (45.79%)	498 (65.10%)	99 (76.15%)	229 (49.59%)
	Unknown	146 (16.63%)	9 (1.18%)	0	19 (4.11%)
**T classification**	T1	253 (28.82%)	167 (21.83%)	33 (25.38%)	150 (32.47%)
	T2	401 (45.67)	466 (60.92%)	76 (58.46)	251 (54.33%)
	T3	70 (7.97%)	95 (12.42%)	15 (11.54%)	28 (6.06%)
	T4	21 (2.39%)	37 (4.84%)	6 (4.62%)	12 (2.6%)
	Unknown	132 (15.03%)	0	0	21 (4.55%)
**N classification**	N0	486 (55.35%)	485 (63.40%)	83 (63.85%)	299 (64.72)
	N1	135 (15.38%)	199 (26.01%)	32 (24.62%)	88 (19.05%)
	N2	106 (12.07%)	64 (8.37%)	15 (11.54%)	52 (11.26%)
	N3	2 (0.22%)	10 (1.31%)	0	0
	Unknown	148 (16.86%)	0	0	23 (4.98%)
**M classification**	M0	483 (55.01%)	625 (81.70%)	129 (99.23%)	NA
	M1	37 (4.21%)	12 (1.57%)	0	NA
	Other	357 (40.66%)	129 (16.86%)	1 (0.77%)	NA
**Tumor stage**	Stage I	409 (46.58%)	380 (49.67%)	73 (56.15%)	NA
	Stage II	76 (8.66%)	235 (30.72%)	34 (26.15%)	NA
	Stage III	118 (13.44%)	131 (17.12%)	23 (17.69%)	NA
	Stage IV	38 (4.33%)	12 (1.57%)	0	NA
	Unknown	10 (1.14%)	7 (0.92%)	0	NA
**Tobacco smoking**	Ever	622 (70.84%)	730 (95.42%)	120 (92.31)	301 (65.15%)
**History**	Never	108 (12.3%)	22 (2.88%)	4 (3.08%)	49 (10.61%)
	Unknown	148 (16.86%)	13 (1.70%)	6 (4.62%)	112 (24.24%)
**Number pack years**	<30	174 (19.82%)	122 (15.95%)	NA	NA
**Smoked**	>=30	327 (37.24%)	499 (65.23%)	NA	NA
	Unknown	377 (42.94%)	144 (18.82%)	NA	NA
**Radiation therapy**	Yes	87 (9.9%)	70 (9.15%)	NA	NA
	No	556 (63.33%)	534 (69.80%)	NA	NA
	Unknown	235 (26.77%)	161 (21.05%)	NA	NA
**Chemotherapy**	Yes	NA	NA	NA	NA
	No	NA	NA	NA	NA
	Unknown	NA	NA	NA	NA
**Additional pharmaceutical**	Yes	97 (11.05%)	58 (7.58%)	NA	NA
**Therapy**	No	117 (13.33%)	152 (19.87)	NA	NA
	Unknown	664 (75.63%)	555 (72.55%)	NA	NA
**Additional radiation**	Yes	97 (11.05%)	55 (7.19%)	NA	NA
**Therapy**	No	120 (13.67%)	151 (19.74%)	NA	NA
	Unknown	661 (75.28%)	559 (73.07%)	NA	NA
**Vital status**	Alive	477 (54.33%)	405 (52.94%)	67 (51.54%)	207 (44.81%)
	Dead	274 (31.21%)	360 (47.06)	62 (47.69%)	236 (51.08%)
	Unknown	127 (14.46%)	0	1 (0.77%)	0

## Data Availability

All data collected for this study are publicly available.

## Supplementary Materials

The following supporting information can be downloaded at: https://www.mdpi.com/article/10.3390/cells11142207/s1, Figure S1: (A), survival analysis of ferroptosis pattern 1 and 2. (B), survival analysis of ferroptosis pattern 1 and 3. (C), survival analysis of ferroptosis pattern 2 and 3. (D), The ferroptosis score comparation between LUAD and LUSCC. (E), The ferroptosis score comparation between EGFR mutation. (F), The ferroptosis score comparation between ALK-eml4 translocation. (G), The comparation our model with existing signatures (PMID: 31417870). (H), TIDE analysis of high and low ferroptosis score groups. (I), Kaplan-Meier plots for OS in the high and low ferroptosis score groups based on LUAD database (https://pubmed.ncbi.nlm.nih.gov/34358469/) (accessed on 2 July 2022). (J), Kaplan-Meier plots for OS in the high and low ferroptosis score groups based on LUSCC database (https://pubmed.ncbi.nlm.nih.gov/32649874/) (accessed on 2 July 2022). ** *p* < 0.01; Table S1: Information of 60 ferroptosis-related genes; Table S2: Mutivariate analysis of the correlation of ferroptosis score with outcomes among LUAD and LUSCC in TCGA cohorts. *, *p* < 0.05. **, *p* < 0.01. ***, *p* < 0.001; Table S3: The list of 86 genes that were differentially expressed among ferroptosis-related gene cluster groups.
