# Supplementary material for: Ferroptosis-Related Genes Are Potential Therapeutic Targets and the Model of These Genes Influences Overall Survival of NSCLC Patients
Source: Cells. 2022 Jul 15;11(14):2207. doi: 10.3390/cells11142207 (PMC9319237; doi:10.3390/cells11142207)
Supplement: Supplementary file 1 [file cells-11-02207-s001.zip › supplementary tables.pdf]

Table S1 Information of 60 ferroptosis-related genes.

Table S1. Information of 60 ferroptosis-related genes				
**Iron Metabolism**	**Lipid Metabolism**	**(Anti)oxidant Metabolism**	**Energy Metabolism**	**Other**
FANCD2	ACSL4	GCLC	GLS2	CD44
NCOA4	AKR1C1	SLC7A11	SLC1A5	HSPB1
TFRC	AKR1C2	KEAP1	GOT1	CRYAB
PHKG2	AKR1C3	NQO1	G6PD	RPL8
HSBP1	ALOX15	ABCC1	PGD	SAT1
ACO1	ALOX5	CHAC1	ATP5G3	TP53
FTH1	ALOX12	GSS		EMC2
STEAP3	CARS	GCLM		AIFM2
NFS1	CBS	NFE2L2		
IREB2	CISD1	NOX1		
HMOX1	CS			
MT1G	DPP4			
	HMGCR			
	GPX4			
	LPCAT3			
	FDFT1			
	ACSL3			
	PEBP1			
	ZEB1			

	SQLE			
	FADS2			
	ACSF2			
	PTGS2			
	ACACA			

Table S2 Mutivariate analysis of the correlation of ferroptosis score with outcomes among LUAD and LUSCC in TCGA cohorts. *, P < 0.05. **, P < 0.01. ***, P < 0.001.

variable	Univariate Cox analysis						Multivariate Cox analysis					
	coef	HR（95%CI）	HR	95%CI	z	P-value	coef	HR（95%CI）	HR	95%CI	z	P-value
TCGA-LUAD（Over Survival）												
pathologic_N(N1 VS N0)	0.87	2.392（1.7000-3.365）	2.39	（1.7000-3.365）	5.01	5.56E-07	1.14E-01	1.121（0.53238-2.360）	1.12E+00	（0.53238-2.360）	0.3	0.76402
pathologic_N(N2 VS N0)	1.11	3.046（2.0843-4.452）	3.05	（2.0843-4.452）	5.75	8.75E-09	-8.93E-02	0.9145（0.31343-2.668）	9.15E-01	（0.31343-2.668）	-0.164	0.87011
pathologic_N(N3 VS N0)	-1.48E+01	0.0000003817（0.0000-Inf）	3.82E-07	（0.0000-Inf）	-0.007	0.994	-1.55E+01	0.0000001811（0.00000-Inf）	1.81E-07	（0.00000-Inf）	-0.007	0.99476
pathologic_N(NX VS N0)	3.49E-01	1.417（0.5194-3.868）	1.42E+00	（0.5194-3.868）	0.681	0.496	3.18E-01	1.374（0.32848-5.747）	1.37E+00	（0.32848-5.747）	0.435	0.66343
pathologic_T(T1a VS T1)	0.5432	1.7215（0.8302-3.570）	1.7215	（0.8302-3.570）	1.46	0.144322	1.03E+00	2.799（1.20113-6.524）	2.80E+00	（1.20113-6.524）	2.384	0.01710 *
pathologic_T(T1b VS T1)	0.1112	1.1176（0.5096-2.451）	1.1176	（0.5096-2.451）	0.278	0.781325	4.33E-01	1.542（0.64942-3.662）	1.54E+00	（0.64942-3.662）	0.982	0.3263
pathologic_T(T2 VS T1)	0.5989	1.8201	1.820	（1.1474-2.8	2.5	0.01095	1.01E	2.753	2.75E	（1.32726-5.	2.7	0.0065

		（1.1474-2.887）	1	87）	44	3 *	+00	（1.32726-5.710）	+00	710）	21	2 **
pathologic_T(T2a VS T1)	0.3602	1.4337 （0.7472-2.751）	1.4337	（0.7472-2.751）	1.083	0.278614	4.35E-01	1.545 （0.67109-3.559）	1.55E+00	（0.67109-3.559）	1.023	0.30642
pathologic_T(T2b VS T1)	0.2666	1.3056 （0.5285-3.225）	1.3056	（0.5285-3.225）	0.578	0.563386	-2.05E-01	0.8144 （0.23931-2.772）	8.14E-01	（0.23931-2.772）	-0.329	0.74252
pathologic_T(T3 VS T1)	1.2252	3.4048 （1.8681-6.205）	3.4048	（1.8681-6.205）	4.001	6.32e-05 ***	1.04E+00	2.818 （0.98572-8.057）	2.82E+00	（0.98572-8.057）	1.933	0.05322 .
pathologic_T(T4 VS T1)	1.2391	3.4525 （1.6779-7.104）	3.4525	（1.6779-7.104）	3.366	0.000763 ***	1.45E+00	4.243 （1.17148-15.368）	4.24E+00	（1.17148-15.368）	2.201	0.02774 *
pathologic_T(TX VS T1)	1.7036	5.4934 （1.2771-23.631）	5.4934	（1.2771-23.631）	2.289	0.022108 *	1.09E+00	2.981 （0.33769-26.309）	2.98E+00	（0.33769-26.309）	0.983	0.32565
radiation_therapy(YES VS NO)	0.7707	2.1613 （1.454-3.213）	2.1613	（1.454-3.213）	3.808	0.00014 ***	5.12E-01	1.669 （1.07454-2.592）	1.67E+00	（1.07454-2.592）	2.28	0.02261 *
tumor_stage.diagnoses(stage ia VS stage i)	-0.003856	0.996151 （0.23670-4.192）	0.9961	（0.23670-4.192）	-0.005	0.9958	2.31E-01	1.26 （0.24016-6.610）	1.26E+00	（0.24016-6.610）	0.273	0.78466
tumor_stage.diagnoses(stage ib VS stage i)	0.152227	1.164425 （0.28058-4.832）	1.1644	（0.28058-4.832）	0.21	0.8339	-9.78E-02	0.9069 （0.18123-4.538）	9.07E-01	（0.18123-4.538）	-0.119	0.90529
tumor_stage.diagnoses(stage ii VS stage i)	2.042505	7.709902 （0.6925-85.836）	7.7099	（0.6925-85.836）	1.661	0.0967						
tumor_stage.diagnoses(stage iia VS stage i)	1.14533	3.143478 （0.73324-13.477）	3.1434	（0.7332-13.477）	1.542	0.123	1.38E+00	3.972 （0.84724-18.620）	3.97E+00	（0.84724-18.620）	1.75	0.08017 .
tumor_stage.diagnoses(stage iib VS stage i)	0.868296	2.382847 （0.56827-9.992）	2.3828	（0.56827-9.992）	1.187	0.2351	5.37E-01	1.71 （0.38265-7.645）	1.71E+00	（0.38265-7.645）	0.703	0.48233
tumor_stage.diagnoses(stage iiia VS stage i)	1.3885	4.008833 （0.96237-16.699）	4.0088	（0.96237-16.699）	1.907	0.0565	1.15E+00	3.149 （0.57935-17.116）	3.15E+00	（0.57935-17.116）	1.328	0.18417
tumor_stage.diagnoses(stage	1.0211	2.776259	2.776	（0.56012-13	1.2	0.2112	1.36E-	1.014	1.01E	（0.11249-9.	0.0	0.9903

iiib VS stage i)	04	（0.56012-13.761）	2	.761）	5		02	（0.11249-9.135）	+00	135）	12	
tumor_stage.diagnoses(stage<br>iV VS stage i)	1.4214<br>12	4.142964<br>（0.94718-18.121）	4.142<br>9	（0.9471-18.<br>121）	1.8<br>88	0.059	8.77E-<br>01	2.404<br>（0.4863-11.886）	2.40E<br>+00	（0.48634-11<br>.886）	1.0<br>76	0.2819<br>8
**Ferroptosis score**	0.0876<br>9	1.09165<br>（1.05-1.135）	1.091<br>6	（1.05-1.135<br>）	4.4<br>06	**1.06E-0<br>5 *****	5.45E-<br>02	1.056<br>（1.0063-1.108）	1.06E<br>+00	（1.00634-1.<br>108）	2.2<br>17	**0.0266<br>2 ***
TCGA-LUSC（Over<br>Survival）												
pathologic_N(N1 VS N0)	0.0647<br>5	1.0669<br>（0.7795-1.460）	1.066<br>9	（0.7795-1.4<br>60）	0.4<br>04	0.686	2.80E-<br>01	1.324<br>（0.65738-2.665）	1.32E<br>+00	（0.65738-2.<br>665）	0.7<br>85	0.4323
pathologic_N(N2 VS N0)	0.2737<br>6	1.3149<br>（0.8285-2.087）	1.314<br>9	（0.8285-2.0<br>87）	1.1<br>62	0.245	5.33E-<br>01	1.704<br>（0.46353-6.267）	1.70E<br>+00	（0.46353-6.<br>267）	0.8<br>03	0.4222
pathologic_N(N3 VS N0)	0.9141<br>6	2.49467<br>（0.6139-10.138）	2.494<br>6	（0.6139-10.<br>138）	1.2<br>78	0.201	-1.41E<br>+01	0.0000007243<br>（0.00000-Inf）	7.24E<br>-07	（0.00000-In<br>f）	-0.0<br>05	0.9958
pathologic_N(NX VS N0)	0.3548<br>2	1.42592<br>（0.3520-5.776）	1.425<br>9	（0.3520-5.7<br>76）	0.4<br>97	0.619	-1.54E<br>+01	0.0000001964<br>（0.00000-Inf）	1.96E<br>-07	（0.00000-In<br>f）	-0.0<br>07	0.9942
pathologic_T(T1a VS T1)	0.3959<br>2	1.485750.6642-3.32<br>4	1.485<br>5	（0.6642-3.3<br>24）	0.9<br>64	0.33513	6.19E-<br>01	1.857<br>（0.71870-4.801）	1.86E<br>+00	（0.71870-4.<br>801）	1.2<br>78	0.2012
pathologic_T(T1b VS T1)	0.0727<br>1	1.07542<br>（0.5140-2.250）	1.075<br>4	（0.5140-2.2<br>50）	0.1<br>93	0.84695	4.46E-<br>01	1.561<br>（0.70477-3.459）	1.56E<br>+00	（0.70477-3.<br>459）	1.0<br>98	0.2723
pathologic_T(T2 VS T1)	0.2472<br>6	1.28051<br>（0.8155-2.011）	1.280<br>5	（0.8155-2.0<br>11）	1.0<br>74	0.28282	1.52E-<br>01	1.164<br>（0.41915-3.234）	1.16E<br>+00	（0.41915-3.<br>234）	0.2<br>92	0.7705
pathologic_T(T2a VS T1)	0.3204<br>3	1.37772<br>（0.8035-2.362）	1.377<br>7	（0.8035-2.3<br>62）	1.1<br>65	0.2441	2.88E-<br>01	1.334<br>（0.48803-3.646）	1.33E<br>+00	（0.48803-3.<br>646）	0.5<br>62	0.5744
pathologic_T(T2b VS T1)	0.5307<br>1	1.70014<br>（0.8043-3.594）	1.700<br>1	（0.8043-3.5<br>94）	1.3<br>9	0.1646	5.24E-<br>01	1.689<br>（0.50755-5.622）	1.69E<br>+00	（0.50755-5.<br>622）	0.8<br>55	0.3928
pathologic_T(T3 VS T1)	0.6757	1.96543	1.965	（1.1694-3.3	2.5	0.01076	8.11E-	2.25	2.25E	（0.60014-8.	1.2	0.2291

	1	（1.1694-3.303）	4	03）	5	*	01	（0.60014-8.437）	+00	437）	03	
pathologic_T(T4 VS T1)	0.92266	2.51597 （1.2829-4.934）	2.5159	（1.2829-4.934）	2.685	0.00725 **	1.23E+00	3.415 （0.61303-19.028）	3.42E+00	（0.61303-19.028）	1.402	0.161
radiation_therapy(YES VS NO)	0.168	1.183 （0.7469-1.874）	1.183	（0.7469-1.874）	0.716	0.474	2.37E-01	1.268 （0.75498-2.130）	1.27E+00	（0.75498-2.130）	0.898	0.3694
tumor_stage.diagnoses(stage ia VS stage i)	-0.7164	0.4885 （0.1169-2.042）	0.4885	（0.1169-2.042）	-0.982	0.3262	-3.38E-01	0.7134 （0.09883-5.150）	7.13E-01	（0.09883-5.150）	-0.335	0.7378
tumor_stage.diagnoses(stage ib VS stage i)	-0.4778	0.6202 （0.1514-2.540）	0.6202	（0.1514-2.540）	-0.664	0.5066	-1.03E-01	0.9022 （0.16713-4.871）	9.02E-01	（0.16713-4.871）	-0.12	0.9048
tumor_stage.diagnoses(stage ii VS stage i)	0.2744	1.3158 （0.1850-9.357）	1.3158	（0.1850-9.357）	0.274	0.784	-1.50E+01	0.0000003007 （0.00000-Inf）	3.01E-07	（0.00000-Inf）	-0.004	0.9965
tumor_stage.diagnoses(stage iia VS stage i)	-0.4717	0.6239 （0.1461-2.665）	0.6239	（0.1461-2.665）	-0.637	0.5243	-3.20E-01	0.7263 （0.13796-3.823）	7.26E-01	（0.13796-3.823）	-0.377	0.7059
tumor_stage.diagnoses(stage iib VS stage i)	-0.4059	0.6664 （0.1606-2.765）	0.6664	（0.1606-2.765）	-0.559	0.5761	-1.80E-01	0.8349 （0.18812-3.706）	8.35E-01	（0.18812-3.706）	-0.237	0.8125
tumor_stage.diagnoses(stage iii VS stage i)	1.6102	5.004 （0.8281-30.238）	5.004	（0.8281-30.238）	1.754	0.0794						
tumor_stage.diagnoses(stage iiia VS stage i)	-0.2221	0.8009 （0.1917-3.346）	0.8009	（0.1917-3.346）	-0.304	0.7608	6.14E-01	0.5412 （0.12128-2.415）	5.41E-01	（0.12128-2.415）	-0.805	0.421
tumor_stage.diagnoses(stage iiib VS stage i)	0.114	1.1207 （0.2417-5.197）	1.1207	（0.2417-5.197）	0.146	0.8842	-1.19E+00	0.3038 （0.03094-2.984）	3.04E-01	（0.03094-2.984）	-1.022	0.3067
tumor_stage.diagnoses(stage iV VS stage i)	0.6685	1.9513 （0.3775-10.087）	1.9513	（0.3775-10.087）	0.798	0.4251	4.49E-01	1.566 （0.25045-9.794）	1.57E+00	（0.25045-9.794）	0.48	0.6314
**Ferroptosis score**	-0.02433	0.97596 （0.9331-1.021）	0.9759	（0.9331-1.021）	-1.061	**1.28E-05** ***	-5.59E-02	0.9457 （0.89263-1.002）	9.46E-01	（0.89263-1.002）	**-1.898**	**0.0377** *

Table S3 86 differentially expressed genes list

86 differentially expressed genes list
AKR1C3
AKR1C1
GPX2
NQO1
AKR1B10
ALDH3A1
GSTA1
ADH1C
TSPAN8
MSMB
LCN2
NTS
SCGB1A1
MT1E
AQP5
MMP28
SLC34A2
PIGR
HPGD
C7
C4BPA

DMBT1
CYP4B1
MMP13
TXNRD1
PGD
G6PD
OSGIN1
TALDO1
GCLM
SLC7A11
CYP4F11
TRIM16
GCLC
ME1
GSR
CABYR
TXN
CYP4F3
UCHL1
NR0B1
CBR3
UGDH
PIR
TSKU
CBR1

MAP2
TSPAN7
KIAA0319
ABCC2
PFN2
ODC1
SPP1
ICAM1
CST6
CDH3
DPP4
TFPI2
CRABP2
ABCB6
PRDX1
RIT1
TKT
ABCC1
ALDH3A2
MEGF9
AKR1C4
TPD52L1
EPHX1
ADH7
ADAM23

HGD
GSTM3
SCN9A
ALDH1A1
ANXA10
FZD10
ABCC3
SERPINB5
CA12
FXYD3
CLDN8
PI3
PKP1
S100P
KRT6A
