# Supplementary figures and images for: Ferroptosis-Related Genes Are Potential Therapeutic Targets and the Model of These Genes Influences Overall Survival of NSCLC Patients
Source: Cells. 2022 Jul 15;11(14):2207. doi: 10.3390/cells11142207 (PMC9319237; doi:10.3390/cells11142207)

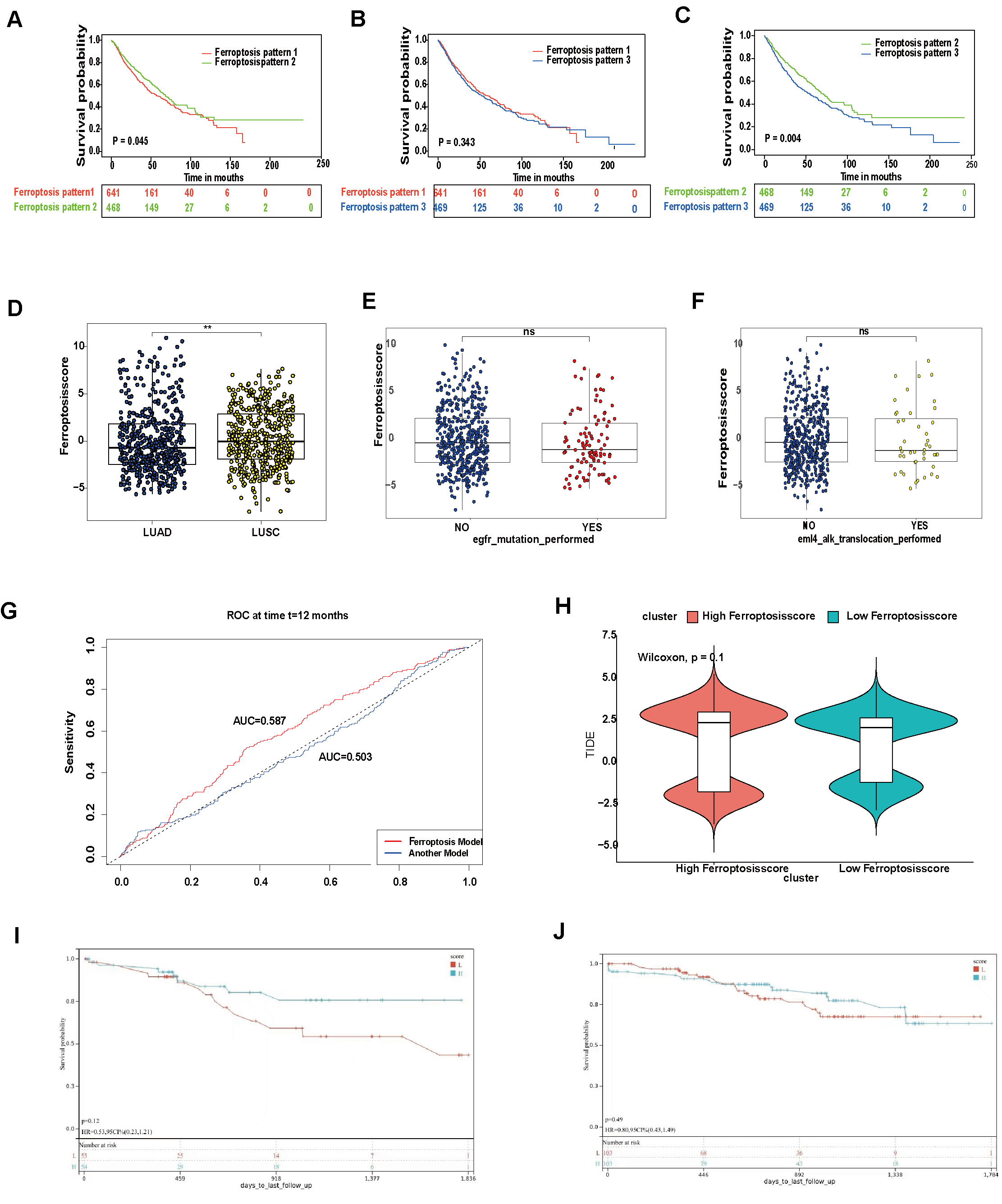

Supplement: Supplementary file 1 [file cells-11-02207-s001.zip › Figure S1.tif]
